# Sexual Function in Women with Breast Cancer: An Evidence Map of Observational Studies

**DOI:** 10.3390/ijerph192113976

**Published:** 2022-10-27

**Authors:** Nelson Rodrigues-Machado, M. Jesús Quintana, Raquel Gómez-Gómez, Xavier Bonfill-Cosp

**Affiliations:** 1 Department of Paediatrics, Obstetrics & Gynaecology and Preventive Medicine, Universtitat Autònoma de Barcelona, 08193 Bellaterra, Spain; 2 Hospital Universitari Vall d’Hebron, 08035 Barcelona, Spain; 3 Institut d’Investigació Biomèdica Sant Pau (IIB Sant Pau), 08041 Barcelona, Spain; 4 Hospital de la Santa Creu i Sant Pau, 08025 Barcelona, Spain; 5 Centro Cochrane Iberoamérica, 08025 Barcelona, Spain; 6 Centro de Investigación Biomédica en Red de Epidemiología y Salud Pública (CIBERESP), 28029 Madrid, Spain

**Keywords:** breast cancer, breast neoplasms, cancer, evidence map, oncology, psychosexual disorders, quality of life, sexual dysfunction, sexual function, women

## Abstract

Breast cancer (BC) is the leading cause of cancer in women, and has implications for sexual function (SF). In this study, we used an evidence map to identify, describe, and organise the current available evidence regarding SF in women with BC. We searched the MEDLINE, PsycINFO, and CINAHL databases for observational studies assessing SF in women with BC published in English, Spanish, Portuguese, and French between 2000 and 2021 (sample ≥ 50 women). Of the 64 included studies (13,257 women with BC), 58 were published since 2010. Women who were married, partnered, or in relationships represented 74.1% of the entire sample. Only a single study was conducted on women representing a sexual minority. We identified 22 assessment instruments and 40 sexual dysfunction (SdF) domains. The number of publications on SF in women with BC has increased in the last 10 years, but still remains low. Some groups of women are underrepresented, and some SdF domains are underdiagnosed, with the assessment instrument used affecting which domains are studied. Women with BC need to be better screened, as their quality of life (QoL) is affected by SdF.

## 1. Introduction

Female breast cancer (BC) is the fifth leading cause of cancer mortality worldwide; it accounts for one in four cancer cases and one in six cancer deaths. In 2020, BC represented 11.7% of all new cancer cases (an estimated 2.3 million new cases). The incidence rates are 88% higher in emerging countries than in transitioned countries, and countries with lower human-development-index (HDI) scores have a 17% higher mortality rate than countries with higher HDI scores [1].

In recent years, BC survival has improved due to early detection strategies and access to timely, effective, and affordable care [2]. However, health providers and health systems need to address BC survivor issues and concerns regarding the treatment and disease course.

In a recent systematic review on adverse mental health outcomes, the researchers revealed that BC survivors have an increased risk of anxiety, depression, neurocognitive dysfunction, sexual dysfunction (SdF), and suicide compared with noncancer groups. The prevalence of SdF, in particular, is reported to range between 20% and 60% [3]. In another study, the researchers reported that women with BC have a high prevalence of SdF (73.4%) and lower average sexual function (SF) scores [4]; the main problems include penetration pain, desire, lubrication, dysfunctional excitement, and reproductive concerns [5,6]. In a study conducted in our hospital to evaluate the social, economic, and professional impacts of BC on women, the researchers found that SF was the most affected quality-of-life (QoL) dimension, and especially during treatment, with a score of 20.7% in the European Organisation for Research and Treatment of Cancer Quality of Life 23-item breast cancer questionnaire [7].

According to the fifth edition of the Diagnostic and Statistical Manual of Mental Health Disorders (DSM-5) [8], SdF covers a heterogeneous group of disorders that are typically characterised by a clinically substantial impairment in a person’s ability to sexually respond or experience sexual pleasure. Female SF, for which researchers have characterised several models in the literature, comprises more than just arousal and orgasm [9].

We need to organise the substantial evidence reported in the scientific literature assessing SF in women who have had BC through a standardised methodology.

The Global Evidence Mapping (GEM) initiative was established in 2007 to provide a general overview of the existing research on traumatic brain and spinal cord injuries [10]. Such evidence maps, which are based on systematic and wide-ranging searches to identify knowledge gaps and future research needs, present results in a user-friendly format (often a visual, graph, or searchable database) [11]. They are both a useful first step in the systematic review process and support for the decision-making process for policy and practice [12,13].

The purpose of our evidence map was to identify, describe, and organise the available evidence regarding SF in women who have BC in order to aid a better understanding of the existing studies, identify the evidence gaps in the literature, and make recommendations for future research.

## 2. Materials and Methods

### 2.1. Study Design

We based this mapping review, which we carried out in accordance with the GEM initiative, on a methodology that involved three core tasks: setting the map boundaries and context; searching for and selecting relevant studies; reporting on the yield and study characteristics [10]. We also followed the Preferred Reporting Items for Systematic Reviews and Meta-Analysis Extension for Scoping Reviews (PRISMA-ScR) recommendations [14]. We designed a protocol and published it in the Open Science Framework (https://osf.io/4qbgu/).

### 2.2. Eligibility Criteria

We considered observational studies with samples of ≥50 women with BC, published between January 2000 and March 2021 in English, Spanish, Portuguese, or French, and evaluating SF using a specific structured measure, as eligible studies. We did not include older published studies, as sexuality is a controversial subject that is influenced by multiple factors and is variable over the years.

### 2.3. Search Strategy

We performed the systematic literature searches for original articles in the following databases: MEDLINE (via PubMed), PsycINFO, and CINAHL. We conducted the last search on 7 March 2021. We used comprehensive controlled vocabulary and free-text terms. The full electronic search strategy for MEDLINE (via PubMed) was as follows: ((“breast neoplasms” [mesh] OR “breast cancer” [ti]) AND (“sexuality” [majr] OR “sexual dysfunction, physiological” [mesh] OR “sexual activit*” [tiab] OR “sexual dysfunction” [tiab] OR “sexual function*” [tiab] OR “sexual interest*” [tiab] OR “sexual desire*” [tiab] OR “sexuality” [tiab] OR “sexual*” [ti])). We adapted the search strategy to the requirements of each database.

### 2.4. Study Selection

We imported the retrieved studies into COVIDENCE reference manager software. We first eliminated duplicate articles, after which two independent researchers screened the titles and abstracts and applied the predefined eligibility criteria to remove ineligible studies. We next eliminated the full texts of the eligible studies to make a final decision regarding the eligibility. We resolved the disagreements in a final online meeting. We clearly justified the reasons for the study exclusions.

### 2.5. Data Extraction

To manage the data and records, we designed a template form in Microsoft Word to individually record the data for each included study. We recorded data on the authors, publication years, journals, study designs, countries, objectives, participants (marital status, sexual orientation, and age recorded as mean/median or range), instruments used to assess sexual function, and domains.

## 3. Results

### 3.1. Selected Studies

**Figure 1:**
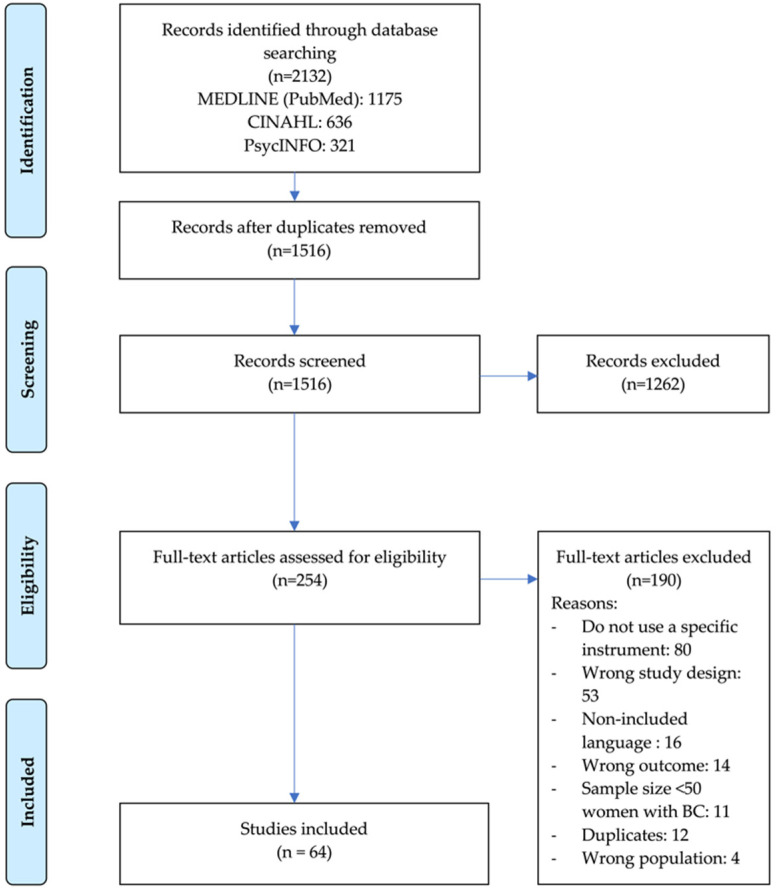
Preferred Reporting Items for Systematic Reviews and Meta-Analysis flow diagram of study selection process.

After we removed the duplicates, we screened a total of 1516 titles and abstracts for eligibility. Of the 254 articles selected for full-text screening, we included 64 that met the eligibility criteria in this review. We present the details on the study inclusion and reasons for exclusion in the flowchart in Figure 1.

### 3.2. Study Characteristics

We present the details of the authors, years of publication, designs, countries, objectives, and participants for the 64 studies in Table 1. The 64 studies, with minimum and maximum sample sizes of 55 and 1957, respectively, included 13,257 women with BC. One study was conducted with 85 lesbian or bisexual women with a female partner, and 51 studies provided data on the mean age, which ranged from 31.4 to 63.4 years. Of the 59 studies (9825 women, 74.1%) that included women who were married, partnered, or in a relationship, in 19 of them, the researchers exclusively focused on women with this profile.

**Figure 2:**
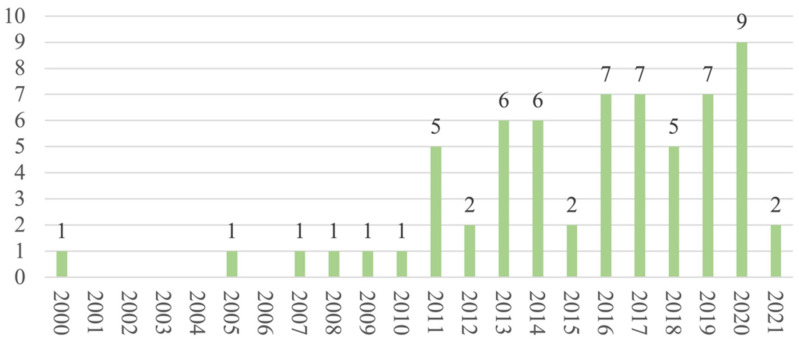
Distribution of articles by year.

**Figure 3:**
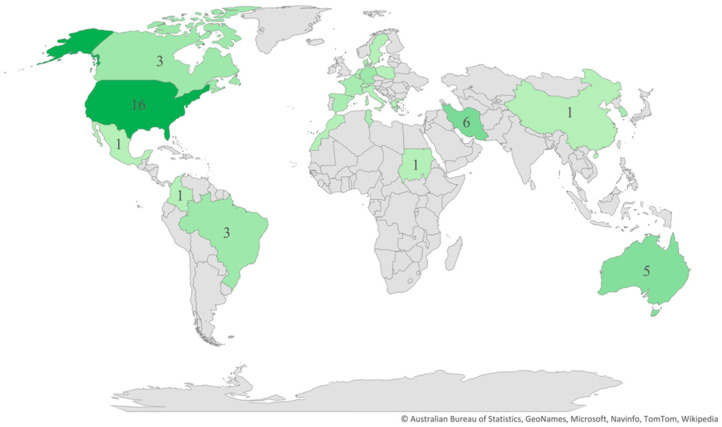
Distribution of articles by country.

Regarding the publication years, most of the studies (*n* = 58) were published between 2011 and 2021 (Figure 2), and most (*n* = 42) were cross-sectional in design. As for the regions and countries, 20 studies were conducted in North America, 16 in Europe, 11 in the Middle East, 5 in Australia, 4 in South America, 4 in Africa, 3 in East Asia, and 1 in South Asia (Figure 3). The USA and Iran were the most represented countries (*n* = 16 and *n* = 6, respectively), followed by Australia and Turkey (*n* = 5 each).

### 3.3. SF Assessment

In most of the studies, the researchers used a single instrument to assess the SF, although six studies used two instruments. We present the summaries of the 22 different instruments that we identified in Table S1. The most frequently used instrument was the Female Sexual Function Index (*n* = 35), followed by the Sexual Activity Questionnaire (*n* = 8), PROMIS Sexual Function and Satisfaction Measures Brief Profile (SexFS) (*n* = 4), and Watts Sexual Function Questionnaire and Cancer Rehabilitation Evaluation System Short Form (CARES-SF) sexual subscale (*n* = 3 each). The instruments used in just a single study were as follows: the Short Sexual Function Scale; Specific Sexual Problems Questionnaire; Golombock–Rust Inventory of Sexual Satisfaction; Questionnaire on Women’s Sexual Function; MacCoy Female Sexuality Questionnaire; Sexual Complaint Screener for Women; Sexual Function Questionnaire; Arizona Sexual Experience Scale; Short Form of the Questionnaire for Screening Sexual Dysfunction; Changes in Sexual Function Questionnaire; Sexual Quotient–Female Version; Sexual Interest and Desire Inventory–Female; Sexual Functioning Questionnaire–Women; Short Form of the Personal Experience Questionnaire; 10-item Menopausal Sexual Interest Questionnaire; 28-item Sexual Function Questionnaire; Relationship and Sexuality Questionnaire.

We identified a total of 40 independently assessed domains, as follows: desire and satisfaction (*n* = 41 each); orgasm (*n* = 40); lubrication (*n* = 39); arousal (*n* = 37); pain (*n* = 36); pleasure (*n* = 8); discomfort and interest (*n* = 6 each); frequency (*n* = 5); vaginal discomfort (*n* = 4); relationship (*n* = 3); vaginismus, excitation, habit, vulvar discomfort–labial, and vulvar discomfort–clitoral (*n* = 2 each). Other domains were evaluated only in a single study (swelling of the labia; reduced length of the vagina; reduced elasticity of the vagina; communication; avoidance; touch; anticipatory anxiety; sexual initiative; masturbation problems; activity; sexual drive; sexual aversion; importance; tiredness; sexual attractiveness; feeling; responsibility; libido; partner problems; responsiveness; sexual enjoyment; distress).

In 32 studies, researchers report data on the relationship between SdF and age, which is associated with older age groups (*n* = 14), younger age groups (*n* = 3), and non-associated with age (*n* = 15). The type of treatment was negatively related to SdF, whether it was mastectomy (*n* = 15/29), chemotherapy (*n* = 11/28), or hormone therapy (*n* = 11/30).

## 4. Discussion

On this evidence map, we included 64 studies published in the last 20 years, comprising a total of 13,257 women with BC for whom SdF was assessed. We observed an evident increase in the number of publications over time (58 of the 64 included studies were published in the last 10 years), which suggests a growing interest in understanding how BC impacts women’s SF.

However, given the importance of the issue, we consider the number of publications to be too low. SdF is a common side effect of cancer treatments, and so it is imperative for health providers to routinely include a comprehensive evaluation of sexual health in the workups for such patients from the outset [77]. Female sexuality is a complex issue, as physiological, psychological, and sociocultural factors and interpersonal relationships all play a part, not to mention the fact that the delayed study of this issue may be due to the historical stigmatisation of women and their sexuality [78].

Interestingly, most of the included women were married, partnered, or in relationships, with a substantial number of researchers selecting this status as an inclusion criterion, which suggests that women without partners, who may experience their sexuality in a different way (e.g., masturbation), are underrepresented.

Only one study was conducted with lesbian or bisexual women having a female partner [21]. Researchers have found that SF is independent of sexual orientation, and the fact that sexual minorities appear to have different social attitudes and sexual practices may imply specific consequences of the physical effects of cancer and its treatment for lesbian, gay, and bisexual populations [79,80,81]. The limited literature on SF in women with BC from sexual minorities remains a key aspect of survivor health outcomes that requires further study [82,83].

On our evidence map, we identified 22 validated instruments used by the authors to assess SF in women with BC. Authors used the Female Sexual Function Index in over 50% of the studies (*n* = 35). This brief 19-item multidimensional self-report instrument for assessing the key dimensions of SF in women, which incorporates the criteria of SdF as defined and recognised by international diagnostic systems [8], was designed and validated for use in clinical trials and epidemiological studies [84]. Women with BC found it to be the right length, easy to complete, and relevant to their experience, and it also demonstrates excellent psychometric properties (high internal consistency, test–retest reliability, and evidence of construct validity) [85]. The second most-used instrument was the Sexual Activity Questionnaire, which is rapidly and easily administered and an acceptably reliable measure. The questionnaire describes SF in terms of the levels of sexual activity, pleasure, and discomfort [86], has been demonstrated to be a useful instrument for measuring sexual activity in women with cancer [87], has been validated in different countries, and has good psychometric properties [88,89,90]. The PROMIS Sexual Function and Satisfaction Measures Brief Profile (SexFS) is also widely used, which is another reliable and valid tool for measuring self-reported SF and satisfaction among men and women with cancer. The instrument is comprehensive in scope, covering both physical and psychological constructs, and provides a comprehensive assessment of satisfaction and key SF domains [91]. Although researchers have previously validated all the instruments and they have good psychometric properties, their scopes differ, and they do not evaluate the same domains. Furthermore, some authors present results as a total SF score, overlooking the fact that an individual may have several simultaneous SdF disorders and that diagnosis should be individual [8].

The researchers assessed a total of 40 domains with the 22 assessment instruments. The most studied domains were desire, satisfaction, orgasm, lubrication, arousal, and pain. Some of the domains reflect internationally accepted classifications as reviewed by the Fourth International Consultation on Sexual Medicine [92], with the following considered to reflect SdF: hypoactive sexual desire dysfunction; female sexual arousal dysfunction; female orgasmic dysfunction; female genital–pelvic pain dysfunction; persistent genital arousal disorder; postcoital syndrome; hypohedonic orgasm; painful orgasm. However, several of the domains reflected in our review are not included in international classifications, which indicates a lack of agreement and the need for further work regarding the definitions and diagnostic criteria, given the need to avoid underestimating SdF [93]. No single assessment instrument measured all the reported domains, which is a fact that adds further complexity to the concept of SdF and leads to the underestimation and underdiagnosis of potential SdF in women with BC.

Finally, in some studies, researchers report measures of the relationships between SdF and age, mastectomy, chemotherapy, and hormone therapy. While Park et al. found that older women were more likely to have a poorer QoL than younger women, especially in terms of the items directly related to sexuality [94], other researchers found that younger women experienced more SF problems [95,96]. Some of the heterogeneity in the data may be explained by the age cut-off for younger versus older women, as well as certain sociocultural factors. In relation to the type of treatment, in a systematic review, the authors report that surgical options other than mastectomy have a better impact on the SF of women with BC [97]. In another study, the authors report that hormone therapy has significant side effects for the QoL of women, including SdF, specifically in terms of the loss of sexual interest, vaginal dryness, and pain during sex [98]. The loss of ovarian function and subsequent early menopause, as side effects due to chemotherapy, often cause SdF symptoms [99]. Nonetheless, certain individual features (e.g., culture, religion, and psychological and physical status) may also influence the way women experience BC.

### 4.1. Study Limitations

This evidence map has some limitations. The search was last updated in March 2021, which means that we may have missed studies published after that date. Furthermore, we only searched three databases; however, these databases are comprehensive, and so we consider that this limitation did not substantially affect our main findings. Another limitation was that we only selected specific SF assessment instruments and excluded studies based on general instruments (e.g., QoL instruments) that only partially evaluate certain aspects of SF. Although we recognise the importance of general instruments, we consider that specific measures may be more sensitive for the detection of small changes in this specific concept.

As for the strengths, as far as we are aware, ours may be the first evidence map that provides a comprehensive synthesis of the available evidence on SF in women with BC reported in the last 20 years. Furthermore, we based our evidence map on a standardised methodology that requires a systematic search and enables the results to be presented for easy and user-friendly interpretation.

### 4.2. Clinical Implications

The evidence map methodology used in this study highlights the important knowledge gaps and identifies future research needs. Our review suggests that SF in women with BC is a broad and complex concept. We suggest that health providers, researchers, and policymakers may need to rethink the concept of SF in a more inclusive way, and they should furthermore consider improving the assessment instruments used to date by including every domain, thereby avoiding underdiagnosis. A valid, reliable, interesting, and easy-to-use measurement instrument allows us to more precisely evaluate SF. Although every health system depends on its own principles, culture, and resources, we strongly recommend that clinicians use/introduce the most suitable instrument in their clinical practice to support decision-making and improve the QoL of woman with BC.

## 5. Conclusions

This evidence map provides a broad vision on how the research on SF in women with BC has been conducted so far.

While studies of SF in women with BC have substantially increased in number over the last 10 years, the importance and frequency of this health problem indicate that the number is still too low. Most of the studies include only women who are married, partnered, or in relationships, which leaves single, lesbian, and bisexual women underrepresented, which can be solved with new studies conducted on these specific groups of women.

Although the studies include a significant number of SF-related domains, this very much depended on the specific assessment instrument used, leading to the underestimation and underdiagnosis of some dysfunctions. Therefore, SdF should not be undervalued, as it can cause suffering in these women and might delay recovery. Future research should focus on ways to better screen for SdF in women with BC and improve their QoL.

## Figures and Tables

**Table 1:** Characteristics of studies included in evidence map.

Author, Year	Journal	Study Design	Country	Objective	Participants (Age in Years)
Abasher, 2009 [15]	*Psycho-Oncology*	Case–control	Sudan	To assess whether there is any decrease in sexual function as a result of breast cancer treatment.	100 BC/100 CG (range: 25–55)
Aerts et al., 2014 [16]	*The Breast*	Prospective	Belgium	To prospectively investigate the presence of sexual dysfunctions and the psychological and relational functioning in women after mastectomy and breast-conserving therapy, and to compare the sexual functioning of breast cancer patients with a control group of healthy women.	149 BC/81 CG (mean ME: 54.51/mean BCT: 57.26/mean CG: 56.12)
Alacacioglu et al., 2014 [17]	*Asian Pacific Journal of Cancer Prevention*	Cross-sectional	Turkey	To investigate the anxiety, depression, and sexual satisfaction levels of Turkish breast cancer patients and their partners.	100 BC (mean: 44.7)
Archangelo et al., 2019 [18]	*Clinics*	Cross-sectional	Brazil	To evaluate the impact of breast reconstruction after mastectomy on specific aspects of the patient quality of life, including sexual function, body image, and depression.	90 BC (mean RG: 47.5/mean ME: 48/mean CG: 47)
Assogba et al., 2020 [19]	*Cancers*	Cross-sectional	France	To identify the clinical, social, and economic determinants of health-related quality of life, and to describe other living conditions of young long-term breast cancer survivors.	218 BC (mean: 47.5)
Bober et al., 2013 [20]	*Journal of Sexual Medicine*	Prospective	USA	To characterise sexual functioning in women recently diagnosed with ductal carcinoma in situ, as well as at 9 and 18 months of follow-up.	304 BC (median: 50.3)
Boehmer et al., 2014 [21]	*The Journal of Sex Research*	Case–control	USA	To compare the sexual function of breast cancer survivors (cases) to an age- and partner-status-matched control group of sexual minority women without cancer.	85 BC/85 CG (mean BC: 51.6/mean CG: 50.9)
Brédart et al., 2011 [22]	*Psycho-Oncology*	Cross-sectional	France	To assess the prevalence and associated factors of the sexual activity, sexual problems, and sexual satisfaction of French early-stage breast cancer survivors.	378 BC (mean: 53)
Bueno-Robles et al., 2015 [23]	*Psicooncología*	Cross-sectional	Colombia	To determine the effect of mood, anxiety, and depression on sexual health, and the impact on Colombian women who have undergone treatment for breast cancer.	103 BC (mean: 48)
Cobo-Cuenca et al., 2018 [5]	*Plos One*	Cross-sectional	Spain	To determine whether there are changes in the sexuality of women after breast cancer, to understand the sexual function of women with breast cancer in Spain, and to describe the relationship between the sociodemographic and clinical variables and sexual dysfunction.	514 BC (mean: 46.34)
Cordoba-de Juan et al., 2019 [24]	*Fisioterapia*	Cross-sectional	Spain	To describe sexual function state in women treated for breast cancer over a year after the diagnosis of breast cancer.	109 BC (median: 56.89)
Cornell et al., 2017 [25]	*Annals of Surgical Oncology*	Prospective	USA	To evaluate the trends in sexual function in women with breast cancer from the time of diagnosis to designated follow-up surgery using a validated sexual questionnaire.	226 BC (median: 56)
Cortés-Flores et al., 2017 [26]	*Aesthetic Plastic Surgery*	Cross-sectional	Mexico	To use the Female Sexual Function Index questionnaire to evaluate and compare the sexuality of women who underwent conservative mastectomy, mastectomy alone, and breast reconstruction after cancer treatment in private practice in Mexico.	74 BC (mean CM: 46/mean MRM: 50.1/mean MRMR: 45.2)
Davis et al., 2010 [27]	*The Nurse Practitioner*	Cross-sectional	USA	To better understand the relationship between sexuality and quality of life in women who have undergone surgical treatment for breast cancer.	72 BC (mean: 61.03)
Ellouz et al., 2019 [28]	*Sexologies*	Cross-sectional	Tunisia	To investigate the prevalence of sexual dysfunction in a population of women followed for breast cancer, and the factors associated with it.	100 BC (mean: 42.6)
Elmas et al., 2020 [29]	*Turkish Journal of Oncology*	Prospective	Turkey	To compare the differences in the sexual function of breast cancer patients undergoing breast cancer surgery and modified radical mastectomy followed by chemotherapy and radiation therapy.	71 BC (median: 43)
Farthmann et al., 2016 [30]	*Supportive Care in Cancer*	Prospective	Germany	To evaluate the influence of chemotherapy for breast cancer on women’s health-related quality of life, sexual function, and depression.	79 BC (mean: 47.46)
Fogh et al., 2021 [31]	*Acta Oncologica*	Cross-sectional	Denmark	To explore the prevalence of clinically relevant sexual dysfunction among breast cancer survivors on adjuvant endocrine therapy, determine the associated factors of sexual dysfunction, explore the extent of the distress caused by specific impairments in sexual function, and analyse whether these were perceived as consequences of breast cancer treatment by breast cancer survivors.	333 BC (mean: 58.74)
Fouladi et al., 2021 [32]	*Asian Pacific Journal of Cancer Prevention*	Cross-sectional	Iran	Not stated	144 BC (mean: 31.4)
Frechette et al., 2013 [33]	*Breast Cancer Research and Treatment*	Prospective	Canada	To document changes in the gynaecological symptoms, sexual problems, and sexual dysfunction (as per DMS-IV criteria) among postmenopausal women with early-stage breast cancer over a 6-month period following the initiation of endocrine therapy, and to identify the predictors of sexual dysfunction 6 months after endocrine treatment.	66 BC (mean: 61)
Gambadrella et al., 2018 [34]	*Endocrine*	Cross-sectional	Italy	To evaluate the impact of different treatment strategies and steroid hormone levels on sexual function in 122 breast cancer women.	122 BC (mean: 46.9)
Gandhi et al., 2019 [35]	*American Journal of Clinical Oncology*	Cross-sectional	USA	To assess the associations of the breast cancer surgical modality and adjuvant therapy on women’s sexual dysfunction in survivorship.	278 BC
Harirchi et al., 2012 [36]	*Journal of Experimental & Clinical Cancer Research*	Prospective	Iran	To elucidate the issue and contribute to the existing knowledge on the topic, and to provide necessary information for implementing possible future interventions to improve the quality of life of breast cancer patients.	216 BC (mean: 44.3)
Herbenick et al., 2008 [37]	*Cancer Nursing*	Cross-sectional	USA	To use a reliable and valid measure to examine the sexual function of women younger than 50 years at the time of their breast cancer diagnosis, and to explore their interest in sexual enhancement products.	115 BC (median: 37.8)
İzci et al., 2020 [38]	*European Journal of Breast Health*	Prospective	Turkey	To examine the pretreatment and post-treatment anxiety, depression, and sleep and sexual function levels in patients with breast cancer.	56 BC/52 CG (mean BC: 53/mean CG: 52.5)
Kedde et al., 2013 [39]	*Supportive Care in Cancer*	Case–control	Netherlands	To determine the prevalence of sexual dysfunction in young women with breast cancer, and to assess the relationship between the treatment administered for breast cancer and sexual dysfunction.	332 BC/1430 CG (mean BC: 38.7/CG range: 22–49)
Kowalczyk et al., 2019 [40]	*Clinical Breast Cancer*	Retrospective	Poland	To evaluate the correlates and impact factors of the sexual function, prevalence of sexual dysfunction, quality of sexual life, and body image of female breast cancer survivors.	128 BC (median: 52.5)
Landi et al., 2016 [41]	*Cancer Causes & Control*	Cross-sectional	USA	To examine the association between the use of endocrine therapy and incident urinary incontinence and sexual dysfunction.	548 BC (mean: 58.1)
Lashani et al., 2020 [42]	*Supportive Care in Cancer*	Cross-sectional	Iran	To explore the types and roles of the relationships between the sexual function, sense of coherence, and wellbeing in a sample of Iranian female breast cancer survivors.	181 BC (mean: 47.04)
Lee et al., 2015 [43]	*Psycho-Oncology*	Cross-sectional	South Korea	To examine the changes in the sexual activity and function of younger breast cancer survivors who were sexually active before diagnosis, and to explore the risk factors that have negative impacts on them.	304 BC (median: 46)
Ljungman et al., 2018 [6]	*Psycho-Oncology*	Prospective	Sweden	To investigate the sexual dysfunction and reproductive concerns in women under the age of 40 years at breast cancer diagnosis, and to identify predictors of high levels of problems and potential interdependence between sexual dysfunction and reproductive concerns.	181 BC (mean: 36.5)
Manganiello et al., 2011 [44]	*European Journal of Oncology Nursing*	Cross-sectional	Brazil	To evaluate the sexual functioning of postmasectomy breast cancer patients and its associations with their quality of life, the personal characteristics of women and their partners, breast reconstruction, cancer staging, and adjuvant therapies.	100 BC (-)
Mayer et al., 2019 [45]	*Archives of Gynecology and Obstetrics*	Retrospective	Germany	To analyse the sexual activity, sexual functioning, and quality of life in patients after the completion of treatment for breast cancer and ovarian cancer.	183 BC/62 OC/60 HG (median BC: 56/median OC: 53/median CG: 46)
Metcalfe et al., 2012 [46]	*Annals of Surgical Oncology*	Prospective	Canada	To report on the changes in the psychosocial functioning over 1 year following breast cancer surgery in three groups of women, including those with mastectomy alone, those with mastectomy and immediate reconstruction, and those with delayed reconstruction.	190 BC (mean MA: 53.5/mean MIR: 46.2/mean DR: 51.6)
Notari et al., 2018 [47]	*European Journal of Cancer Care*	Cross-sectional	Switzerland	To fill in a gap in the present literature by describing women’s sexual functioning in the early weeks of active treatment for breast cancer.	75 BC (mean: 52.55)
Oberguggenberger et al., 2017 [48]	*BMC Cancer*	Cross-sectional	Germany	To investigate the self-reported sexual health outcomes of breast cancer survivors in routine after-care in comparison with women with no previous or current breast cancer.	105 BC/97 NBC (mean BC: 49/mean NBC: 49)
Öztürk et al., 2016 [49]	*Japan Journal of Nursing Science*	Cross-sectional	Turkey	To assess the sexual function of Turkish women undergoing a surgical procedure, and to determine whether there were differences between Turkish women undergoing postmastectomy breast reconstruction and those undergoing breast-conserving surgery or mastectomy alone.	100 BC (mean: 47)
Paiva et al., 2016 [50]	*Archives of Sexual Behavior*	Cross-sectional	Brazil	To investigate the prevalence of the sexual dysfunction and identify the associated conditions in a patient population of Brazilian breast cancer survivors, focusing on obesity-related conditions.	216 BC (mean: 51.9)
Park et al., 2013 [51]	*Supportive Care in Cancer*	Cross-sectional	South Korea	To study the relationships among menopausal symptoms, sexual function, depression, and quality of life in women with breast cancer undergoing chemotherapy.	200 BC (mean: 45.64)
Parker et al., 2007 [52]	*Annals of Surgical Oncology*	Prospective	USA	To prospectively examine the short- and long-term effects of mastectomy with reconstruction, mastectomy without reconstruction, and breast-conserving therapy on aspects of psychosocial adjustment and quality of life in a sample of 258 women with breast cancer.	258 BC (mean MWR: 49.2/mean MA: 52.8/mean BCT: 53.7)
Qureshi et al., 2018 [53]	*Aesthetic Surgery Journal*	Cross-sectional	USA	To assess the prevalence of sexual health issues in a plastic surgery patient population including breast cancer survivors and women without breast cancer.	90 BC/149 NBC (mean BC: 51.2/mean NBC: 47)
Raggio et al., 2014 [54]	*Psychology & Health*	Cross-sectional	USA	To assess four self-reported sexual morbidity domains, including sexual function, sexual distress, body change stress, and body satisfaction, in a sample of long-term breast cancer survivors, and to evaluate the influence of select psychosocial and medical factors based on the extant literature, including age, treatment modality (e.g., mastectomy, specific treatment effects (e.g., weight gain and premature menopause), and psychosocial factors (e.g., depression, marital/relationship status and satisfaction, and quality of life)), within and across four sexual morbidity domains.	83 BC (mean: 56.21)
Reese et al., 2020 [55]	*The Journal of Sexual Medicine*	Cross-sectional	USA	To determine, in a sample of BC outpatients, how commonly women sought help for sexual concerns from a healthcare provider, from other individuals, or from alternate sources, and to examine whether the help-seeking was associated with women’s sexual function/activity, self-efficacy for clinical communication about sexual health, or sociodemographic/medical characteristics.	144 BC (mean: 56)
Robinson et al., 2017 [56]	*The Journal of Sexual Medicine*	Cross-sectional	Australia	To document the prevalence of and factors associated with low desire, sexually related personal distress, hypoactive sexual desire dysfunction, and pelvic-floor dysfunction in women 10 years after breast cancer diagnosis.	625 BC (median: 65.1)
Rojas et al., 2017 [57]	*Breast Cancer Research and Treatment*	Cross-sectional	USA	To explore the impact of mastectomy type on sexual function, as measured by the Female Sexual Function Index, satisfaction with appearance, and the reconstructed breast’s role in intimacy.	60 BC (median TMRM: 52/median SSM: 50.57 median NSM: 46.5)
Rosenberg et al., 2020 [58]	*Jama Surgery*	Prospective	USA	To describe the changes in these outcomes from 1 to 5 years following diagnosis comparing bilateral mastectomy vs. breast-conserving surgery and unilateral mastectomy, as well as to examine the differences by primary surgery type, receipt of radiation, and reconstruction.	826 BC (mean BCS: 35.9/mean UM: 36.4/mean BM: 36.1)
Rottmann et al., 2017 [59]	*Acta Oncologica*	Longitudinal	Denmark	To examine whether individual and partner sexual functioning, affectionate behaviour, emotional closeness, and depressive symptoms are associated with change over time in the satisfaction with the sex lives of sexually active heterosexual couples dealing with BC, and to explore whether the associations differ between patients and partners.	287 BC (mean: 55.77)
Rowland et al., 2000 [60]	*Journal of the National Cancer Institute*	Cross-sectional	USA	To examine the characteristics of women undergoing lumpectomy, mastectomy with reconstruction, and mastectomy alone, and the relationship of the different surgical treatments to specific aspects of the health-related quality of life, body image, and physical and sexual functioning.	1957 B (mean MWR: 50.3/mean LG: 55.9/mean MA: 58.9)
Safarinejad et al., 2013 [61]	*Psycho-oncology*	Cross-sectional	Iran	To compare sexual function, self-esteem, and quality of life in young women with breast cancer by lumpectomy with those from the age-matched general female population.	186 BC/204 CG (mean BC: 37.7/mean CG: 37.2)
Sayakhot et al., 2011 [62]	*Menopause*	Cross-sectional	Australia	To investigate menopausal symptoms, psychological symptoms (including anxiety and depression), sexual function, and body image experienced by younger Australian women with breast cancer, and to examine the effect of different breast cancer treatments (chemotherapy, adjuvant endocrine therapy, and ovariectomy) on the menopausal, physical, and psychological symptoms.	114 BC (mean: 47.2)
Sbitti et al., 2011 [63]	*BMC Women’s Health*	Cross-sectional	Morocco	To prospectively evaluate the body image scale and impact of breast cancer therapy on the sexuality of women from Morocco.	120 BC (mean: 45.3)
Schover et al., 2014 [64]	*The Journal of Sexual Medicine*	Cross-sectional	USA	To provide a more detailed picture of the sexual problems during the first two years on aromatase inhibitor therapy, to provide a benchmark for a prospective intervention trial.	129 BC (mean: 63.4)
Shandiz et al., 2016 [65]	*Asian Pacific Journal of Cancer Prevention*	Cross-sectional	Iran	To investigate sexual function and factors affecting it in women with breast cancer.	94 BC (mean: 45.20)
Soldera et al., 2018 [66]	*Breast Cancer Research and Treatment*	Prospective	Canada	To evaluate sexual health in long-term breast cancer survivors compared with aged-matched controls, determine the impact of chemotherapy and endocrine therapy on the sexual functioning, and compare the related symptoms, such as the gynaecological, vasomotor, and bladder complaints, between groups to potentially explain the source of any differences in sexual function.	248 BC/159 CG (mean BC: 62/mean CG: 59)
Sorouri et al., 2019 [67]	*Archives of Psychiatry Research*	Cross-sectional	Iran	To compare negative emotions, body image, sexual schemas, and sexual function in women with breast cancer after mastectomy and healthy women.	105 BC/100 CG (mean BC: 41.09/mean CG: 41.5)
Speer et al., 2005 [68]	*The Breast Journal*	Case–control	USA	To investigate how testosterone levels, mood, body image, depression, relationship quality, and age influence the sexual function of female BCSs who have been treated with surgery, radiation, chemotherapy, or adjunctive hormone therapy.	55 BC (mean: 53.4)
Tahir et al., 2020 [69]	*Psycho-Oncology*	Cross-sectional	Pakistan	To explore the mediating role of body image (dissatisfaction) between sexual functioning (SF) and marital intimacy in Pakistani women with breast cancer.	118 BC (mean: 39.58)
Tucker et al., 2020 [70]	*Supportive Care in Cancer*	Cross-sectional	Australia	To compare the sexual function and quality of life in female breast cancer survivors with and without a history of bilateral salpingo-oophorectomy.	172 BC (mean BSO: 53/mean no BSO: 58)
Tucker et al., 2016 [71]	*The Breast*	Cross-sectional	Australia	To investigate the prevalence of the sexual dysfunction in women with a prior history of breast cancer following risk-reducing salpingo-oophorectomy (RRSO), and to compare this to women without a previous diagnosis of breast cancer. The secondary objectives were to describe the effects of mastectomy, breast reconstruction, and antioestrogen therapy on the sexual function and quality-of-life outcomes of women with a previous diagnosis of breast cancer after RRSO.	60 BC (mean: 50)
Usta et al., 2017 [72]	*International Journal of Caring Sciences*	Cross-sectional	Turkey	To determine the frequency of sexual dysfunction and factors affecting sexual dysfunction in women with breast cancer receiving chemotherapy.	118 BC (mean: 47.74)
Vaidakis et al., 2014 [73]	*European Journal of Gynaecological Oncology*	Prospective	Greece	To record how the treatment of breast and gynaecological cancer may affect the subjective perception of the interpersonal relation of the couple with respect to female sexuality. The authors also attempted to associate and evaluate disorders of desire, arousal, orgasm, and pain, as well as problems of the sexual relationship, with cancer diagnosis and treatment.	67 BC (mean: 50.5)
Webber et al., 2011 [74]	*The Oncologist*	Prospective	Australia	To examine the incidence of sexual problems after breast cancer in a real-world setting, the predictors of sexual problems over 12 months after adjuvant therapy, and the potential impact of sexual problems on the quality of life.	92 BC (mean: 49.8)
Yuan et al., 2020 [75]	*Supportive Care in Cancer*	Cross-sectional	China	To identify the unobserved distinct latent classes/subgroups of breast cancer patients in China with respect to various sexual health measures, and to examine the association of the latent membership with the individual characteristics.	123 BC (mean: 42.80)
Zaied et al., 2013 [76]	*Bulletin du Cancer*	Cross-sectional	Tunisia	To evaluate the frequency and type of sexual dysfunction in 100 patients treated for nonmetastatic breast cancer in post-treatment monitoring in external consultation (Department of Oncological Medicine, CHU Farhat Hached, Sousse), and to identify the predictive factors of these disorders.	100 BC (mean: 47.24)

BC: breast cancer; BCT: breast cancer therapy; BCS: breast-conserving surgery; BM: bilateral mastectomy; BSO: bilateral salpingo-oophorectomy; CM: conservative mastectomy; CG: control group; DR: delayed reconstruction; LG: lumpectomy group; MA: mastectomy alone; ME: mastectomy; MIR; mastectomy with immediate reconstruction; MRM: modified radical mastectomy; MRMR: modified radical mastectomy with reconstruction; MWR: mastectomy with reconstruction; NBC: no breast cancer; NSM: nipple-sparing mastectomy; OC: ovarian cancer; RG: reconstruction group; SSM: skin-sparing mastectomy; TMRM: total/modified radical mastectomy; UM: unilateral mastectomy; USA: United States of America.

## Data Availability

All the data generated or analysed during this study are included in the published review article.

## Supplementary Materials

The following supporting information can be downloaded at: https://www.mdpi.com/article/10.3390/ijerph192113976/s1, Table S1: Assessment instruments used.
