# Supplementary material for: Sexual Function in Women with Breast Cancer: An Evidence Map of Observational Studies
Source: Int J Environ Res Public Health. 2022 Oct 27;19(21):13976. doi: 10.3390/ijerph192113976 (PMC9654538; doi:10.3390/ijerph192113976)
Supplement: Supplementary file 1 [file ijerph-19-13976-s001.zip › ijerph-1960418-supplementary.pdf]

Table S1. Assessment instruments used

Instruments	Nº Items	Domains	Characteristics
Female Sexual Function Index	19	Desire, excitement, lubrication, orgasm, satisfaction, and pain.	Overall score <26.5 indicates SdF. For each subscale, a score <3.9 is considered as a deterioration on that scale.<br>Validity has been established in a variety of patient populations, including BC.
Sexual Activity Questionnaire	10	Frequency, pleasure, discomfort, and habit.	Higher scores indicating greater pleasure, discomfort, and frequency.<br>Internal consistency between 0.74 and 0.82.
PROMIS Sexual Function and Satisfaction Measures Brief Profile	12	Sexual interest, vaginal lubrication, vaginal discomfort-clitoral, vulvar discomfort-labial, orgasmic function-ability, orgasmic function-pleasure, and satisfaction with sex life.	Higher scores for all domains (except discomfort) signify better function.<br>There is evidence of adequate validity and reliability.
Watts Sexual Function Questionnaire	17	Sexual desire, arousal, orgasm, and satisfaction.	Each item scores on a 5-point Likert scale ranging from 1 "never" to 5 "always". Higher scores indicate more positive SF.<br>It has been used with a variety of chronically ill populations.
Cancer Rehabilitation Evaluation System Short Form Sexual Subscale	3	Attractiveness, interest, and frequency.	Scores for each subscale are calculated from the mean of ratings for each individual item and range from 0 to 4, with higher scores indicating of more problems.<br>Has excellent reliability and validity.
Short Sexual Function Scale	4	Decrease sexual desire, dry vagina, and orgasmic dysfunction.	Four-point scale ranging from 0 (not or doubtfully present) to 3 (extremely present)<br>Internal consistency (Cronbach's α=0.92)
Specific Sexual Problems Questionnaire	Not Stated	Reduced swelling of the labia during arousal, superficial and deep dyspareunia, abdominal pain during intercourse, reduced length and elasticity of the vagina and reduced intensity of orgasm.	Four-point scale ranging from 0 (not or doubtfully present) to 3 (extremely present).<br>Internal consistency (Cronbach's α=0.94).
Golombock-Rust Inventory of Sexual Satisfaction	28	Frequency, orgasmic disorder, vaginismus, lack of communication, avoidance, nonsensuality, intensity, dissatisfaction.	A score of 5 or more points in any category indicates SdF.<br>A validation and reliability study was published elsewhere.

Instruments	Nº Items	Domains	Characteristics
Questionnaire on Women's Sexual Function	14	Desire, excitation or arousal, penetration pain, satisfaction, lubrication, orgasm, and anticipatory anxiety, sexual initiative, and communication	Likert scale with 5 options (1 to 5). Cut points for the range depends on the subscale. Internal consistency of 0.895-0.897 and reliability of 0.597-0.743.
MacCoy Female Sexuality Questionnaire	19	Sexual interest, satisfaction with sexual activity, vaginal lubrication, orgasm, sex partner.	Higher scores indicate more positive feelings. Cronbach's α=0.77 and 2-week test-retest correlations for individual items range from 0.69 to 0.95.
Sexual Complaint Screener-Women	10	Distress.	Present when an impairment in sexual function was experienced as "a considerable problem" or "a very great problem". Good validity when compared to FSFI.
Sexual Function Questionnaire	30	Interest, desire, arousal, orgasm, satisfaction, relationship, behaviour, masturbation, and problems.	Uses a Likert scale and scores range from 0 to 5. Higher scores on the overall scale and subscales indicate better SF. The instrument has established reliability and validity with bone marrow transplant and normative controls.
Arizona Sexual Experience Scale	5	Sexual drive, psychological excitation, vaginal lubrication, capacity to reach orgasm, and satisfaction level after orgasm.	Likert scale where each question scores from 1 to 6. Total score varies from 5 to 30. Low scores show that sexual response is strong, easy, and satisfactory. Hight scores refer to the existence of SdF. Turkish acceptability and reliability works have been applied to the patients with end-stage renal failure.
Short version of the Questionnaire for Screening Sexual Dysfunction	Not stated	Hypoactive sexual desire disorder, sexual aversion disorder, female sexual arousal disorder (subjective arousal disorder, genital arousal disorder), female orgasmic disorder, dyspareunia, and secondary vaginismus.	Five-point or 7-point scale depending on the subscale. Scores at least 3 on the prevalence scale (or 4 on the sexual aversion prevalence items) and 3 on the distress scale is considered SdF.
Changes in Sexual Function Questionnaire	Not stated	Pleasure, desire/frequency, desire/interest, arousal/excitement, and orgasm/completion.	A higher score indicates better SF.
Sexual Quotient-Female Version	10	Sexual satisfaction and functioning.	Scores in a scale from 0 to 5. Final score between 0 to 100. Higher scores indicate better SF. Internal consistency reliability was 0.84.
Sexual Interest and Desire Inventory-Female	13	Sexual desire, SdF, sexual behaviour, and sexual relationship.	Low scores indicate low SF. Internal consistency of 0.925.

Instruments	Nº Items	Domains	Characteristics
Sexual Functioning Questionnaire-Women	21	Interest, desire, activity, excitement, orgasm, satisfaction, relations, masturbations, and problems.	Higher scores indicate better SF. Cronbach's α=0.94.
Short Form of the Personal Experience Questionnaire	Not stated	Feeling, responsibility, frequency, libido, dyspareunia, partner problems.	A cut-off score of 7 or below indicates SdF.
10-item Menopausal Sexual Interest Questionnaire	10	Desire, responsiveness (orgasm and pleasure), and satisfaction	Subscales and an overall score are calculated
28-item Sexual Function Questionnaire	28	Female sexual arousal disorder, female orgasm disorder, hypoactive sexual desire disorder, pain disorder, sexual enjoyment, and relations quality.	Each item is rated on a 5-point Likert scale
Relationship and Sexuality Questionnaire	19	Sexual dysfunction, frequency, and sexual fear.	Likert scale with scores from 0 to 3 or 0 to 4

BC: breast cancer; SF: sexual function; SdF: sexual dysfunction; FSFI: female sexual function index
